# Interactions between mitoNEET and NAF-1 in cells

**DOI:** 10.1371/journal.pone.0175796

**Published:** 2017-04-20

**Authors:** Ola Karmi, Sarah H. Holt, Luhua Song, Sagi Tamir, Yuting Luo, Fang Bai, Ammar Adenwalla, Merav Darash-Yahana, Yang-Sung Sohn, Patricia A. Jennings, Rajeev K. Azad, Jose' N. Onuchic, Faruck Morcos, Rachel Nechushtai, Ron Mittler

**Affiliations:** 1 The Alexander Silberman Institute of Life Science, Hebrew University of Jerusalem, Edmond J. Safra Campus at Givat Ram, Jerusalem, Israel; 2 Department of Biological Sciences and BioDiscovery Institute, University of North Texas, Denton, Texas, United States of America; 3 Center for Theoretical Biological Physics and Departments of Physics and Astronomy, Chemistry and Biosciences, Rice University, Houston, Texas, United States of America; 4 Departments of Biological Sciences and Bioengineering, University of Texas at Dallas, Richardson, Texas, United States of America; 5 Department of Chemistry & Biochemistry, University of California at San Diego, La Jolla, California, United States of America; 6 Department of Mathematics, University of North Texas, Denton, Texas, United States of America; 7 The Wolfson Institute for Applied Structural Biology, Hebrew University of Jerusalem, Edmond J. Safra Campus at Givat Ram, Jerusalem, Israel; Weizmann Institute of Science, ISRAEL

## Abstract

The NEET proteins mitoNEET (mNT) and nutrient-deprivation autophagy factor-1 (NAF-1) are required for cancer cell proliferation and resistance to oxidative stress. NAF-1 and mNT are also implicated in a number of other human pathologies including diabetes, neurodegeneration and cardiovascular disease, as well as in development, differentiation and aging. Previous studies suggested that mNT and NAF-1 could function in the same pathway in mammalian cells, preventing the over-accumulation of iron and reactive oxygen species (ROS) in mitochondria. Nevertheless, it is unknown whether these two proteins directly interact in cells, and how they mediate their function. Here we demonstrate, using yeast two-hybrid, *in vivo* bimolecular fluorescence complementation (BiFC), direct coupling analysis (DCA), RNA-sequencing, ROS and iron imaging, and single and double shRNA lines with suppressed mNT, NAF-1 and mNT/NAF-1 expression, that mNT and NAF-1 directly interact in mammalian cells and could function in the same cellular pathway. We further show using an *in vitro* cluster transfer assay that mNT can transfer its clusters to NAF-1. Our study highlights the possibility that mNT and NAF-1 function as part of an iron-sulfur (2Fe-2S) cluster relay to maintain the levels of iron and Fe-S clusters under control in the mitochondria of mammalian cells, thereby preventing the activation of apoptosis and/or autophagy and supporting cellular proliferation.

## Introduction

The human NEET proteins mitoNEET (mNT) and NAF-1 (encoded by the CISD1 and CISD2 genes, respectively) play major roles in the regulation of apoptosis, autophagy and iron and reactive oxygen species (ROS) homeostasis in cells [1–6]. They contain labile 2Fe-2S clusters, coordinated by 3Cys and 1His ligands, as well as the signature CDGSH motif [7–9]. mNT is localized to the outer mitochondrial membrane and NAF-1 to the ER and the outer mitochondrial membrane [2]. Both mNT and NAF-1 have been implicated in a number of human pathologies including diabetes, neurodegeneration, myocardial injury and cancer, as well as in development, differentiation and aging [10–15].

Gain and loss of function analysis of mNT and NAF-1 in cancer cells revealed that overexpression of mNT or NAF-1 protein promotes cancer cell proliferation [13–15], while suppression of either mNT or NAF-1 protein expression via shRNA decreases cancer cell proliferation and tumor growth [4, 5, 14]. Suppression of mNT or NAF-1 protein expression also results in the over-accumulation of iron and ROS in the mitochondria of cancer cells and the activation of autophagy [4] and apoptosis [5]. Interestingly, mitochondrial iron and ROS over-accumulation, as a consequence of mNT or NAF-1 suppression, could be reversed in cells by treatment with the iron chelator deferiprone (DFP), suggesting that mNT and NAF-1 function in cells is primarily mediated via their effect on iron and/or Fe-S metabolism [4–6]. Overexpression of mNT also protects cells from oxidative stress and ferroptosis [16], whereas overexpression of NAF-1 protects cancer cells from oxidative stress and apoptosis [14]. Moreover, the function of NAF-1 in protecting cancer cells from oxidative stress and promoting cellular proliferation is dependent on the degree of lability of the NAF-1 2Fe-2S cluster during oxidative stress [14]. Thus, when overexpressed in cancer cells, a NAF-1 mutant with a 25-fold more stable 2Fe-2S cluster (H114C) was unable to promote cellular proliferation and protect cells from oxidative stress and apoptosis [14]. Furthermore, potentially functioning as a dominant negative inhibitor of NAF-1 function, overexpression of this NAF-1 variant (H114C) suppressed the growth of xenograft tumors to a level similar to that obtained with shRNA suppression of NAF-1 [14]. The studies described above suggest that NAF-1 and mNT use their labile 2Fe-2S clusters to mediate different redox or cluster transfer reactions that help cancer cells alleviate some of the toxic effects of iron and ROS over-accumulation during oxidative challenge in the mitochondria. This function could be regulated by mNT or NAF-1 in many different cells types [6, 16–18], and represent one of the major functions of these proteins in mammalian and even plant cells [2].

The interaction of mNT and NAF-1 with various cellular proteins was studied in an attempt to determine their mode of function in cells. MitoNEET was found to interact with anamorsin [19] and cytosolic aconitase [20], two Fe-S proteins involved in iron-sulfur biogenesis and iron regulation, as well as with glutathione reductase [21], a redox regulator, and glutamate dehydrogenase 1 [22], a key metabolic enzyme and an insulin regulator. NAF-1 was found to interact with BCL-2, a key protein involved in the regulation of apoptosis and autophagy [3, 23], as well as with CAPN2, a Ca^2+^-activated protease involved in apoptosis activation [24] and anamorsin [19]. The protein-protein interaction studies described above suggest that NAF-1 could have a more pronounced regulatory role in cells, whereas mNT might have a more metabolic- and/or redox/Fe-related role in cells. Despite these possible differences, suppressing mNT or NAF-1 expression in cancer cells resulted in a similar phenotype of over-accumulation of iron and ROS in mitochondria and activation of autophagy [4]. These findings suggest that NAF-1 and mNT could function in the same cellular pathway in cancer cells. To test this possibility we used a combination of cell biology, omics and protein-protein interaction techniques to determine whether NAF-1 and mNT interact and function in the same pathway in cancer cells.

## Results

### Yeast two hybrid (Y2H) analysis using mNT as a bait identified NAF-1 as a potential mNT interaction partner

To identify putative protein-protein interactors of mNT in cancer cells we conducted a comprehensive Y2H screen [25–30] for mNT. As a bait we used the coding sequence for *Homo sapiens*-mitoNEET [aa 31–108; gi:374671792] cloned into pB29 as an N-terminal fusion to LexA. As a prey we used a random-primed human breast tumor epithelial cells cDNA library constructed in pP6. Using a mating approach we then screened over 100 million clones [10 fold the complexity of the library] and selected 176 His+ colonies for further analysis. Prey fragments of the positive clones were amplified by PCR and sequenced at their 5’ and 3’ junctions. The resulting sequences were then used to identify the corresponding interacting proteins in the GenBank database (NCBI). A confidence score (Predicted Biological Score; PBS) was attributed to each interaction as previously described [28]. The top 12 clones with the highest confidence score are shown in Table 1. These include NAF-1 (CISD2), a non-lysosomal calcium-activated protease Calpain-1 (CAPN1), that could be related to CAPN2, previously shown to bind NAF-1 [24], and a number of other proteins that are known to be involved in immune response (e.g., integrin-beta), cytoskeleton (e.g., filamin and mucin) and cancer (e.g., FAT tumor suppressor and UXT). Based on our previous findings suggesting that mNT and NAF-1 could function in the same molecular pathway [4], we validated and studied the putative interaction between mNT and NAF-1 *in vivo* and *in vitro* as described below.

**Table 1:** Yeast two-hybrid analysis of human epithelial breast cancer proteins that interact with mitoNEET.

Interaction Level	Gene Name	Role
A	FLNA	Filamin A, alpha (FLNA). Anchoring and remodeling of the cytoskeleton
		important for cell shape and migration.
A	ITGB5	Integrin- beta 5. Interacts with PTK2, Annexin A5 and PAK4.
A	UXT	Ubiquitously expressed. Also known as androgen receptor
		trapped clone 27 (ART-27) protein. Involved in tumorigenesis as
		it is abundantly expressed in tumor tissues especial prostate.
B	CAPN1	Calpain-1 catalytic subunit. Calcium-activated neutral proteases,
		nonlysosomal, intracellular cysteine proteases
B	NAF-1	CISD2. 2Fe-2S CDGSH iron-sulfur protein.
B	CSGALNACT2	Chondroitin Sulfate N-Acetylgalactosaminyltransferase 2 Elongation
		during chondroitin sulfate synthesis.
B	FAT	FAT tumor suppressor homolog
B	ITGB1	Integrin beta-1. An integrin unit associated with very late antigen
		receptors.
B	MUC5B	Mucin 5B, Oligomeric Mucus/Gel-Forming.
C	GRN	Granulin. Secreted, glycosylated peptide. Granulin family members are
		important in normal development, wound healing, and tumorigenesis.
C	NOTCH3	Type I membrane protein notch. Affects differentiation, proliferation and
		apoptotic programs.
C	SPINT1	Serine Peptidase Inhibitor, Kunitz Type 1

Corresponding interacting proteins were identified by a BLAST search of the GenBank database (NCBI). Confidence score for interaction level (A, B, C; Predicted Biological Score) was attributed to each interaction as previously described (28).

### *In vivo* bimolecular fluorescence complementation (BiFC) analysis of the interaction between mNT and NAF-1 in cancer cells

**Fig 1:**
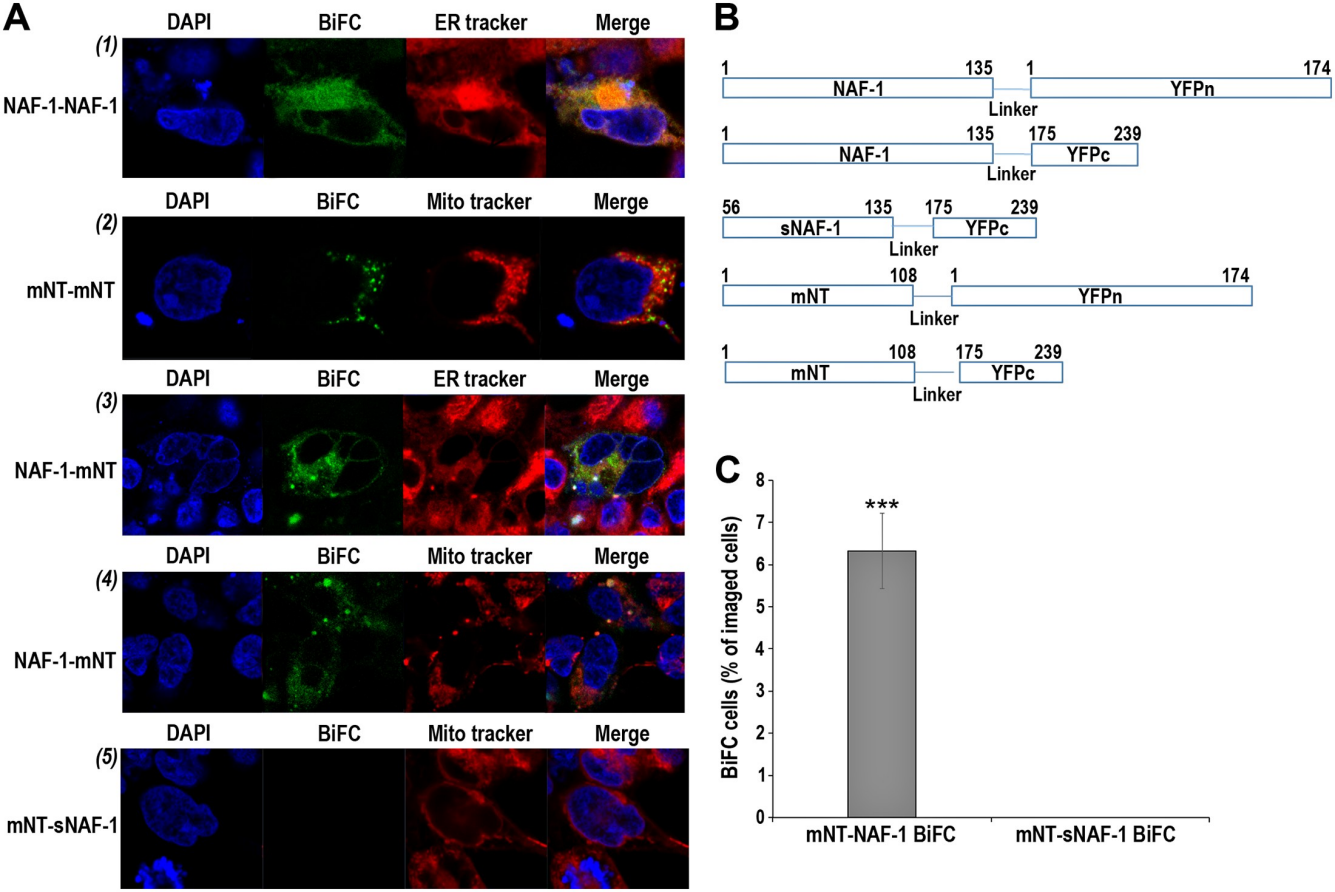
BiFC analysis of mNT-NAF-1 interaction. **A**. Representative images of: *(1)* Positive control for NAF-1 homodimer interaction using co-expression of NAF-1-YFPc and NAF-1-YFPn with ER tracker localization. *(2)* Positive control for mNT homodimer interaction using co-expression of mNT-YFPc and mNT-YFPn with mitochondrial (Mito) tracker localization. *(3)* mNT-NAF-1 interaction following co-expression of NAF-1-YFPc and mNT-YFPn with ER tracker localization. *(4)* mNT-NAF-1 interaction following co-expression of NAF-1-YFPc and mNT-YFPn with Mito tracker localization. *(5)* Negative control for mNT-NAF-1 interaction using co-expression of mNT-YFPn and soluble sNAF-1-YFPc with mitochondrial (Mito) tracker localization. **B**. The different split-YFP/mNT/NAF-1/sNAF-1 vectors used for the in vivo analysis of mNT-NAF-1 interaction shown in A. **C**. A comparison between the BiFC signal obtained with the mNT-NAF-1 interaction (co-transfection with NAF-1-YFPc and mNT-YFPn; mNT-NAF-1) and the BiFC signal obtained with the mNT-sNAF-1 interaction (co-transfection with sNAF-1-YFPc and mNT-YFPn; mNT-sNAF-1). Vector construction, transfection and imaging are described in Materials and Methods. ***, p≤0.001.

To determine whether mNT and NAF-1 interact *in vivo* in human cells we conducted a split-yellow fluorescence protein (YFP) BiFC analysis for their interaction using human embryonic kidney-293 (HEK-293) cells (Fig 1 and S1 Fig). For this purpose the full length clone of NAF-1 (gi:92345) was cloned in frame (C-terminal fusion) to one fragment of YFP (aa 175–239 of YFP; NAF-1-YFPc) and the full length clone of mNT was cloned in frame (C-terminal fusion) to the other fragment of YFP (aa 1–174 of YFP; mNT-YFPn). For the purpose of having a positive control for the split-YFP interaction, and because both mNT and NAF-1 are homo-dimers, we also generated a NAF-1-YFPn and a mNT-YFPc clones. In addition, for the purpose of having a negative control, we generated a soluble NAF-1-YFPc clone that lacked the NAF-1 membrane-anchoring domain (aa 56 to 135; sNAF-1-YFPc). All vectors used in our study are shown in Fig 1B. As shown in Fig 1A, when NAF-1-YFPc and NAF-1-YFPn (NAF-1 positive control) were transiently co-expressed in HEK-293 cells, an ER-localized BiFC positive signal was detected in cells, indicating that NAF-1-NAF-1 dimers could be detected in cells using the split-YFP assay. A similar BiFC signal was detected for mNT-YFPc and mNT-YFPn (mNT positive control) co-transient expression in HEK-293 cells, but this signal co-localized with mitochondria. Interestingly, when NAF-1-YFPc and mNT-YFPn were co-transiently expressed in HEK-293 cells (mNT-NAF-1 interaction), a BiFC signal was detected localized to both the ER and mitochondria (Fig 1A and S1 Fig). These findings indicate that NAF-1 and mNT interact in human cells *in vivo* and that mNT-NAF-1 interactions could occur on both the ER and mitochondria. This result is highly interesting because it suggests that mNT could be mobilized to the ER upon interactions with NAF-1. Furthermore, the presence of the NAF-1 membrane anchoring domain appears to be important for the interaction and localization of the mNT-NAF-1 complex because sNAF-1, that did not contain this domain (negative control), failed to interact with mNT under our experimental conditions (Fig 1 and S1 Fig). Our findings with the Y2H and BiFC assays strongly support an interaction between NAF-1 and mNT in cancer cells and strengthen the Y2H results presented in Table 1. To further examine whether the interaction of mNT and NAF-1 in cells could have implications related to 2Fe-2S cluster transfer reactions, we used native gel assays to study cluster transfer reactions between these two proteins.

### *In vitro* analysis of mNT-NAF-1 interaction reveals that mNT can donate its 2Fe-2S cluster to NAF-1

**Fig 2:**
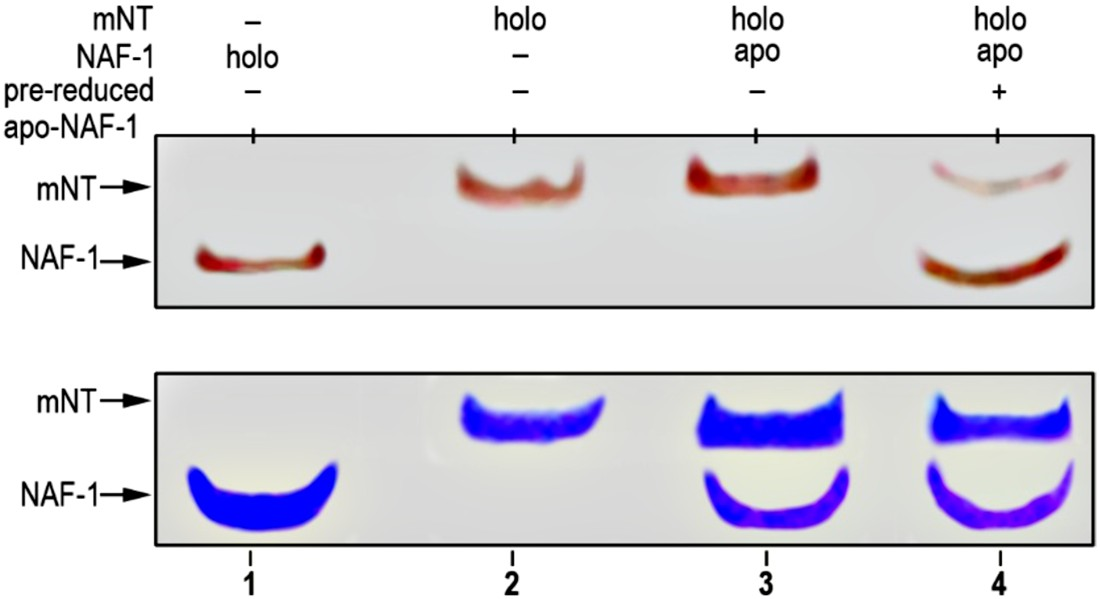
2Fe-2S cluster transfer from holo-mNT to apo-NAF-1. Apo-NAF-1 was incubated at 37°C for 20 min with β-mercaptoethanol and holo-mNT, and chromatographed on a native gel as described in [31] with the modification described in Materials and Methods. Red-colored bands in the upper native gels are indicative of the [2Fe–2S] cluster presence in the two proteins. Blue-colored bands in the lower duplicate gel are the result of Coomassie Blue staining to confirm the presence and levels of the two proteins.

Both NAF-1 and mNT are capable of donating their clusters to apo-acceptor proteins [8, 9, 19, 31]. Our findings that mNT and NAF-1 interact (Table 1; Fig 1) highlight the possibility that, in addition to interacting, they could also transfer their clusters from one to the other and participate in different cluster relay pathways. To test this possibility we purified NAF-1 and mNT as previously described [7–9, 31, 32] and used native gels to study cluster transfer between them. Using a similar approach, we previously demonstrated cluster transfer from NAF-1 or mNT to apo-acceptor proteins such as anamorsin and ferredoxin [8, 9, 19, 31]. Because 2Fe-2S cluster biogenesis is primarily conducted in the mitochondria, wherein mNT is localized [33–36], we reasoned that mNT would be the first to obtain the cluster (from the mitochondria) and transfer it to NAF-1. As shown in Fig 2, oxidized mNT was indeed able to donate its cluster to pre-reduced apo-NAF-1, demonstrating cluster transfer between mNT and NAF-1. This cluster transfer reaction did not occur when apoNAF-1 was oxidized (Fig 2), indicating that the Cys-SH ligands must be reduced to accept and coordinate the 2Fe-2S cluster, similar to what was shown for the transfer of the 2Fe-2S cluster of mNT or NAF-1 to other apo-acceptor proteins such as anamorsin and ferredoxin [8, 9, 19, 31]. Despite repeated attempts, we were unable to observe a similar cluster transfer from holo-NAF-1 to apo-mNT (not shown). This observation could reflect the differences in structure between mNT and NAF-1 that could make the backbone of NAF-1 more rigid at the cluster binding site, and/or differences in the energy level between the two protein forms [2]. Further studies are needed to address these possibilities.

To begin deciphering the interactions between mNT and NAF-1 at the molecular level, we examined whether mNT and NAF-1 harbor an evolutionary-conserved binding interface between them.

### Direct coupling analysis revealed an evolutionary-conserved binding interface between mNT and NAF-1

**Fig 3:**
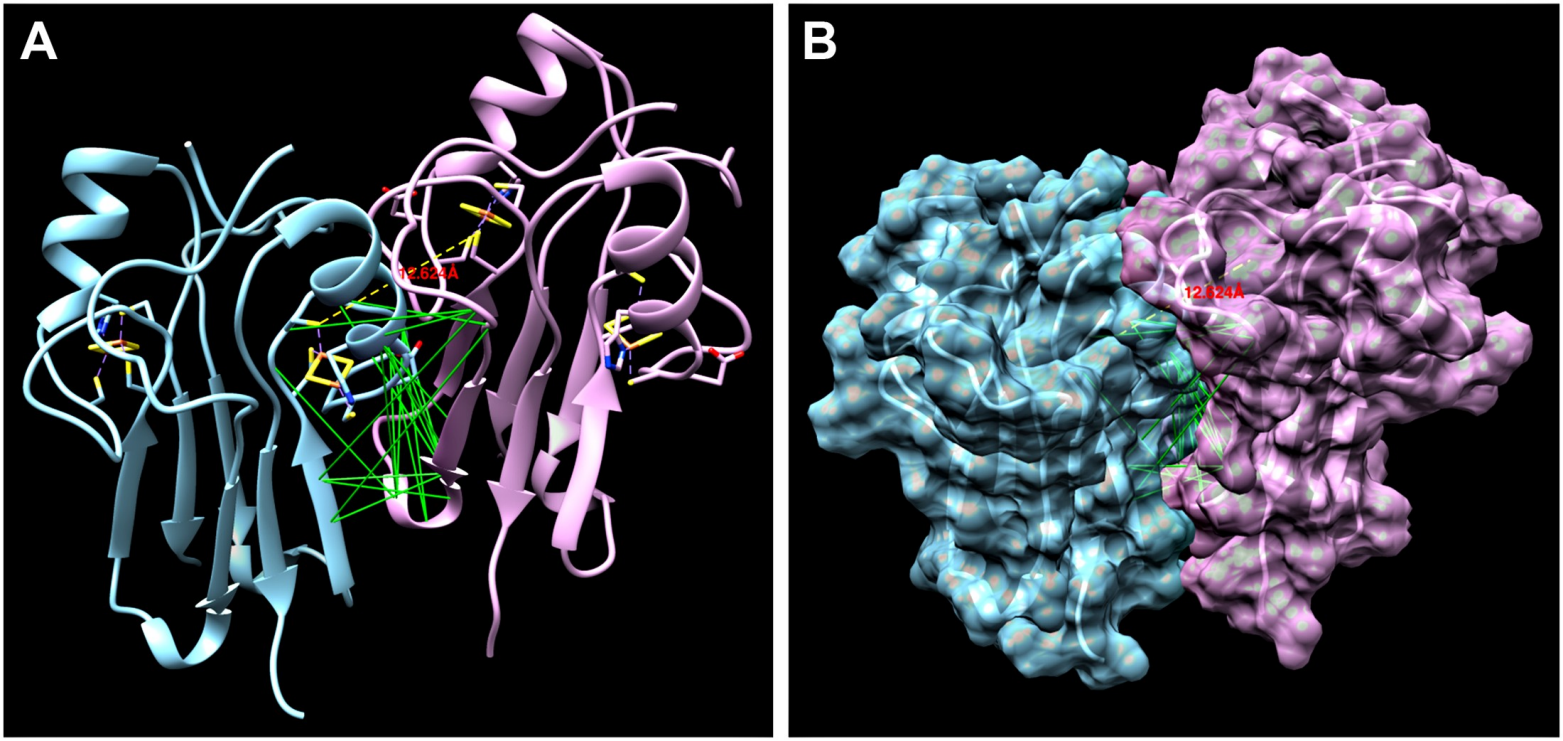
DCA analysis and complex model of NAF-1-mNT interaction. **A**. A cartoon representation of the mNT-NAF-1 complex, with mNT on the left (blue) and NAF-1 on the right (pink). This figure was generated by aligning the PDB crystal structures of mNT (PDB 2QH7) and NAF-1 (PDB 3FNV) with the result of the simulation. The distance between the iron-sulfur clusters is shown to be about 12.6 Å (highlighted in red). **B**. Surface representation of the mNT-NAF-1 complex demonstrating the close fit between the two proteins. A closer view of this lock-and-key part of the interface is shown in S3 Fig. The DCA couplings are depicted as green lines. A full list of the couplings, along with their approximate distances is included in S1 Table.

To build an atomic-level model of mNT-NAF-1 interaction, we studied the co-evolution of amino acid sequences in the family of mNT/NAF-1 using direct coupling analysis (DCA). DCA is a statistical inference framework used to identify amino acids that are coupled through an evolutionary process [37]. We performed DCA on 1130 amino acid sequences of the protein family zf-CDGSH (PF09360) of which both mNT and NAF-1 are members. DCA was previously used to generate highly accurate models of monomeric structures and their conformational diversity [38, 39], as well as complexes and other dimeric interactions [23, 40, 41]. While the methodology has been validated for generating homodimers [40], our current data suggests a tetrameric interaction between mNT and NAF-1 with potentially different tetrameric interfaces compared to those found in homodimers. To account for this difference, we updated our complex modeling protocol based on coevolution and molecular dynamics to identify regions of interaction that are conserved through evolution but not present in homodimeric interfaces. In addition to excluding the signals from co-evolution that contribute to the monomeric fold and those that are not solvent accessible, the known homodimeric contacts were also not considered, leaving only a concise cluster of solvent accessible couplings that are potential tetrameric signals. We selected the top 20 remaining couplings (S2 Fig), ranked based on direct information (DI) values, for predicting the interaction sites between mNT and NAF-1. The DI values for each potential inter-residue pair, obtained using DCA, provided a metric of the most probable residue—residue interactions. For each of the monomers, we mapped the sites in the multiple sequence alignments (MSA) of the protein family zf-CDGSH (PF09360) onto the respective residues of the crystals of mNT and NAF-1. Since mNT transfers an iron-sulfur cluster to NAF-1 (Fig 2), we assume that the location of the cluster in each of the proteins must be at a distance that can facilitate cluster transfer. Previously reported distances that allow iron-sulfur cluster transfer between different proteins are usually within the range of 12Å [42–44]. Therefore, in addition to the co-evolutionary constraints, we added an extra constraint to favor cluster transfer at a distance of ~12 Å. This constraint is important since the co-evolutionary analysis is not designed to capture ligand interactions. Using these constraints as guidelines, a complex was obtained using molecular dynamics to bring the molecules together as described in Materials and Methods and in [40]. The resulting model of the mNT-NAF-1 complex that fully satisfied both the evolutionary coupling and cluster distance constraints is shown in Fig 3 (S2 to S4 Figs). The distance between the two iron-sulfur cluster sites is ~12.6 Å, consistent with previous studies [42–44]. Furthermore, the majority of the evolutionary coupling distances (shown in green) are within 10 Å (see S1 Table), providing further support for the model. Moreover, these couplings all appear within a single interface. The identification of this evolutionary-conserved contact plane between NAF-1 and mNT further supports the interaction between these two proteins in cells. A few of the more distant DCA couplings can be explained by the fact that they are located in the region around the iron-sulfur cluster. This provides further evidence for the cluster’s importance in the interaction between mNT and NAF-1. Qualitatively, the structures appear to be complimentary; the grooves and ridges of mNT fit smoothly with those of NAF-1 (see S3 Fig). Because Fe-S proteins could exhibit some degree of plasticity when transferring clusters [45, 46], mNT and NAF-1 could undergo slight conformational changes during the formation of their complex, which was not accounted for by our model. Our findings that mNT and NAF-1 interact in cells and could mediate cluster transfer reactions (Table 1, Figs 1–3), coupled with our previous analysis of cells with suppressed expression of mNT or NAF-1 (4), support a model in which mNT and NAF-1 function in the same cellular pathway to attenuate iron and ROS levels in the mitochondria. To determine whether mNT and NAF-1 function in the same pathway, or have redundant functions, we tested whether suppressing the expression of mNT, NAF-1, or mNT and NAF-1 would result in a similar effect on mitochondrial iron and ROS levels.

### Double shRNA suppression of mNT and NAF-1 [mNT(-)/NAF-1(-)]] does not result in a significantly larger impairment in mitochondrial membrane potential (MMP) or labile iron and ROS over-accumulation in cancer cells, compared to mNT(-) or NAF-1(-) single suppressed lines

**Fig 4:**
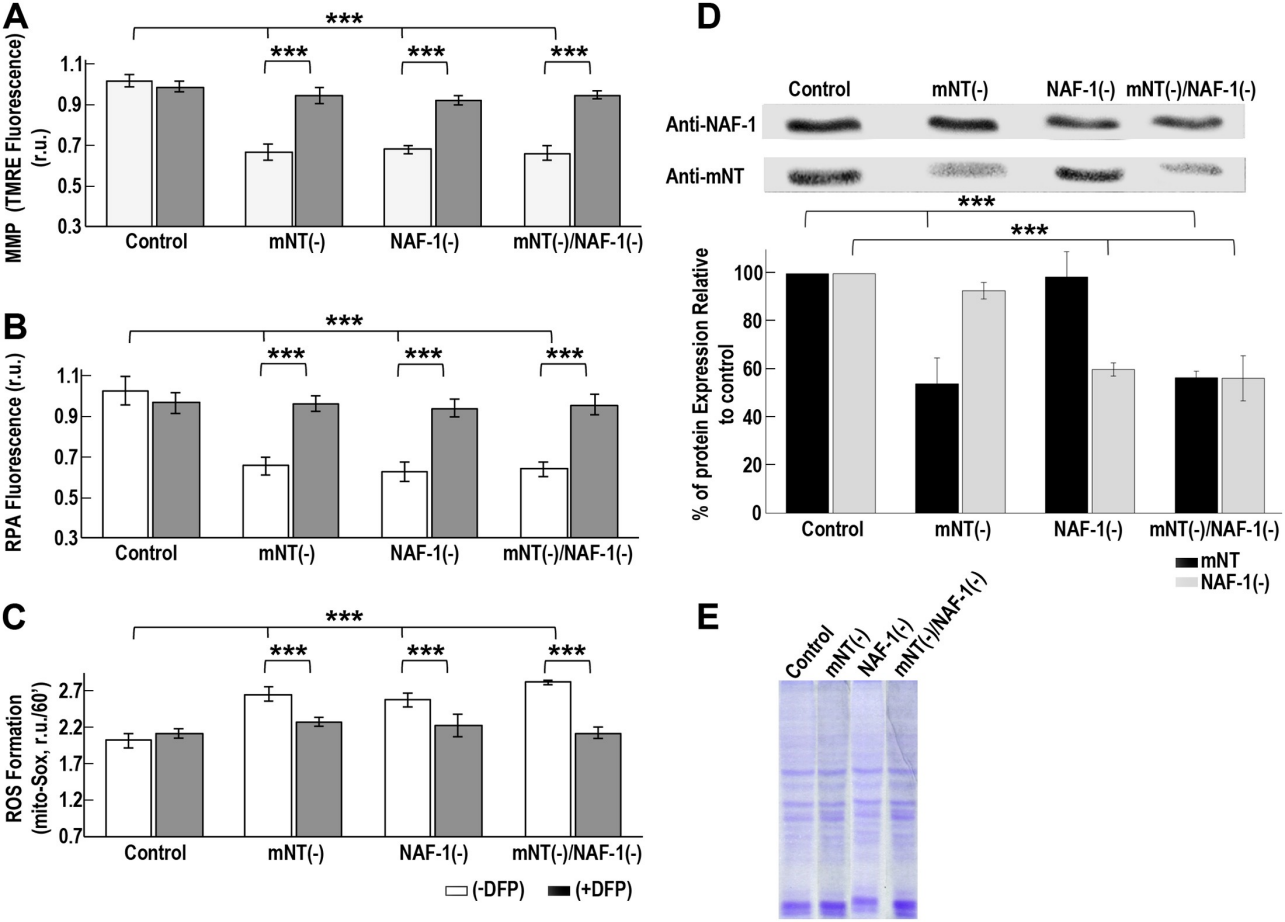
Mitochondrial membrane potential (MMP), labile iron and ROS measurements in cancer cell lines with suppressed mNT and/or NAF-1 expression. Compared to single suppression of mNT [mNT(-)] or NAF-1 [NAF-1(-)], double shRNA suppression of mNT and NAF-1 [mNT(-)/NAF-1(-)] stable lines do not result in a significantly larger impairment in MMP **(A)**, mitochondrial labile iron **(B)** and mitochondrial ROS **(C)** accumulation. The iron chelator DFP (100 μM) is shown to recover the effect of mNT and/or NAF-1 suppression on MMP, mitochondrial labile iron and ROS in all lines in a similar manner **(A-C)**. All measurements were compared to control MDA-MB-231 cell lines transfected with the same shRNA vector containing a scrambled RNA. The expression levels of the mNT and NAF-1 proteins in all lines are shown **(D)**, and Coomassie blue loading controls in **(E)**. ***P <0.001.

Suppression (shRNA) of mNT [mNT(-)] or NAF-1 [NAF-1(-)] expression in human epithelial breast cancer cells (MCF-7 or MDA-MB-231) resulted in the disruption of MMP, the over-accumulation of labile iron and ROS in mitochondria and the activation of autophagy [4]. These results suggest that mNT and NAF-1 are both required for maintaining iron and ROS levels under control in mitochondria of cancer cells. To determine whether mNT and NAF-1 function in the same, or different pathways to mediate these functions in cancer cells we generated double shRNA cell lines with suppressed expression of both mNT and NAF-1. As shown in Fig 4, mNT(-)/NAF-1(-) double suppressed human epithelial breast cancer cell lines did not display a significantly larger impairment in their MMP, or a higher over-accumulation of labile iron or ROS in their mitochondria compared to NAF-1(-) or mNT(-) single suppressed lines. Furthermore, the iron chelator DFP was able to correct all three mitochondrial phenotypes (MMP, iron and ROS) to a similar level in both the signal and double suppressed lines (Fig 4). The findings presented in Fig 4 suggest that mNT and NAF-1 function in the same pathway, that they cannot physiologically compensate for the lack of each other, and that their function in cells is directly linked to the levels of labile iron in mitochondria. To determine the degree of overlap between the pathways altered in cells with suppressed mNT or NAF-1 expression, we conducted RNA-Seq analysis on these lines.

### RNA-Seq analysis reveals a high degree of overlap between transcripts altered in expression in mNT (-) cancer cells and those altered in expression in NAF-1 (-) cancer cells

**Fig 5:**
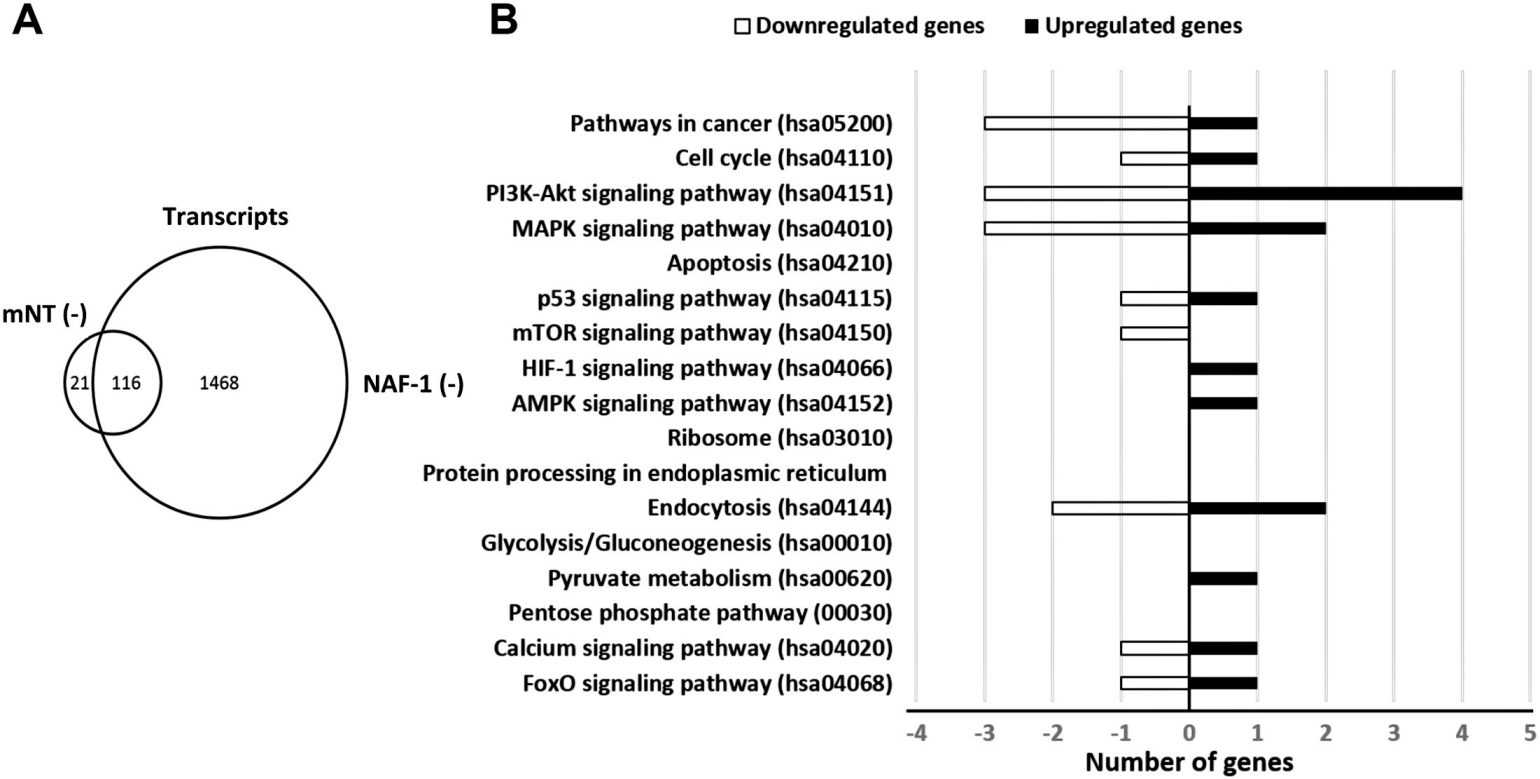
Overlap between transcripts altered in cancer cells with suppressed mNT [mNT(-)] or NAF-1 [NAF-1(-)] expression. **A**. Venn diagram showing the overlap between transcripts altered (p≤0.05) in cancer cells with suppressed mNT [mNT(-)] or NAF-1 [NAF-1(-)] expression detected with RNA-seq analysis. **B**. KEGG annotation of transcripts common to cells with suppressed mNT [mNT(-)] or NAF-1 [NAF-1(-)] expression.

To further examine the degree of overlap between NAF-1 and mNT function in cancer cells we compared the transcriptome profile (RNA-Seq) of human epithelial breast cancer cells with suppressed NAF-1 expression [5] to that of human epithelial breast cancer cells with suppressed mNT expression (conducted side-by-side with the analysis reported in [5] and reported here). For this analysis we compared the transcriptome of control (scrambled vector) cancer cells with that of cancer cells with suppressed (shRNA) NAF-1 or mNT expression grown under the same conditions reported in [5] and sampled side-by-side. As shown in Fig 5A, suppression of NAF-1 expression resulted in the altered (enhanced or suppressed) expression of 1584 transcripts [5]. In contrast, suppression of mNT expression resulted in a moderate response with only 137 transcripts displaying altered expression (enhanced or suppressed; Fig 5A; S2 and S3 Tables). Out of the 137 transcripts altered in expression in mNT(-) cells, 116 were also altered in expression in NAF-1(-) cells (Fig 5A). This high degree of overlap between the transcripts altered in mNT(-) and NAF-1(-) suggests that the majority of responses induced by mNT suppression are also induced by NAF-1 suppression and that these transcripts may hint to the similar function these two proteins are mediating in cancer cells. A KEGG annotation analysis of these common transcripts identified several pathways involved in the regulation of cell cycle and cellular proliferation including MAPK and PI3K-Akt (Fig 5B). Both of these pathways were previously linked to ROS and iron homeostasis in cells, as well as identified and studied in NAF-1(-) cells [47–49]. The findings that suppression of NAF-1 or mNT resulted in similar alterations in these pathways suggest that interactions between mNT and NAF-1, that could involve cluster transfer between the mitochondria and the cytosol, may be involved as one of the signals that trigger or alter these pathways and control cellular proliferation.

## Discussion

**Fig 6:**
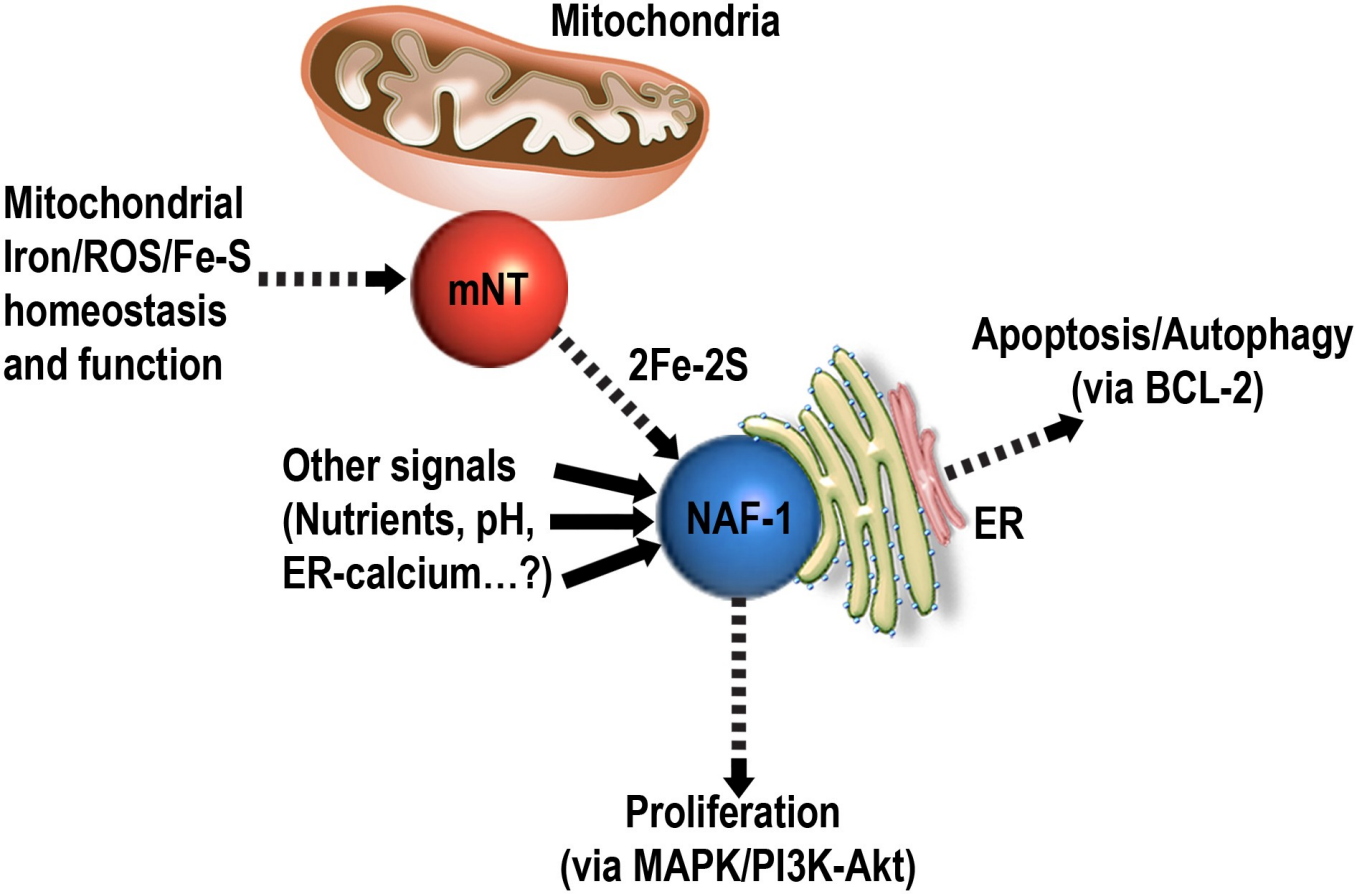
A hypothetical model for the interaction of mNT with NAF-1 in cells. mNT is shown to accept Fe-S clusters from the mitochondria and transfer them to NAF-1. The flow of clusters from mNT to NAF-1 is shown to be used by NAF-1 to regulate different processes such as apoptosis/autophagy activation, as well as cellular proliferation. The cluster relay between mNT and NAF-1 is proposed to link mitochondrial Iron/ROS/Fe-S homeostasis and function with the regulation of cell death/proliferation by the NAF-1/BCL-2/MAPK/PI3K-Akt pathways.

Fe/Fe-S levels are tightly regulated in cells to prevent the accumulation of ROS that could lead to the activation of cell death pathways such as apoptosis and ferroptosis [50]. This process is thought to be highly critical for rapidly proliferating cancer cells that maintain a higher than normal levels of ROS and iron in their mitochondria, while suppressing apoptosis and promoting cellular proliferation [51–53]. Because NAF-1 and mNT were shown to play a critical role in promoting cellular proliferation and maintaining iron and ROS homeostasis in cells [4–6], these two proteins, and especially their clusters, could be used as key targets for the development of anticancer drugs [14, 41]. Despite their very similar function and biochemical features [2], NAF-1 and mNT are not redundant in cells and suppressing the expression of either one of them results in a similar phenotype of mitochondrial iron and ROS over-accumulation and autophagy activation [4]. Here we report that mNT and NAF-1 could interact in cells and that mNT could transfer its clusters to NAF-1 (Figs 1–3; Table 1). These findings could provide an explanation as to why the function of mNT and NAF-1 is not completely redundant in cells, and suggest that mNT and NAF-1 function in the same cellular pathway maintaining mitochondrial labile iron levels under control (Figs 4–6). The site of mNT and NAF-1 interaction, i.e., the mitochondria and the ER (Fig 1 and S1 Fig), suggests that mNT could transfer a 2Fe-2S cluster that it normally accepted from a mitochondrial donor to NAF-1 that is primarily localized on the ER. Our computational model of the complex depicting the dimerization of mNT and NAF-1 (Fig 3) provides further evidence that a cluster transfer occurs between these two proteins. Based on evolutionary conserved signals, the model places the 2Fe-2S cluster holding sites 12.6 apart, which is an appropriate distance to facilitate the exchange of this cluster [42–44]. Residues surrounding the cluster are also highly coupled, indicating that the cluster plays a role in the formation of the complex.

NAF-1 was previously shown to interact at the ER with BCL-2, a key protein involved in autophagy and/or apoptosis regulation [3, 23]. Furthermore, the interaction of NAF-1 with BCL-2 is thought to be dependent on the presence or absence of the NAF-1 cluster. Thus, holo-NAF-1 could bind BCL-2 and prevent autophagy activation, whereas apo-NAF-1 does not bind BCL-2 and does not prevent autophagy activation [3]. Because the ER-localized interaction of NAF-1 with BCL-2 is thought to be dependent on the presence or absence of the 2Fe-2S cluster of NAF-1 [3], the transfer of a cluster from mNT to NAF-1 could be used by cells to monitor the levels of Fe-S or Fe in the mitochondria and to activate autophagy or apoptosis by BCL-2 (Fig 6). Thus, when NAF-1 is suppressed [4, 5], or when NAF-1 does not accept a 2Fe-2S cluster from mNT (Fig 2; [4]), NAF-1 fails to interact with BCL-2 and autophagy and/or apoptosis are activated. In support of this possibility is the finding that, when overexpressed in cancer cells, a mutant of NAF-1 that cannot accept or donate a 2Fe-2S cluster (H114C) fails to regulate mitochondrial iron and ROS levels in cancer cells, and causes enhanced apoptosis activation as well as suppressed cellular proliferation and tumor growth [14]. Further studies are of course required to substantiate the model presented in Fig 6.

Our findings that, compared to cell lines with suppression of mNT or NAF-1 expression, cell lines with double suppression of mNT and NAF-1 expression do not display a significantly larger impairment in MMP, or accumulate higher levels of mitochondrial Fe and ROS (Fig 4), coupled with the findings that the iron chelator DFP can rescue all of these phenotypes in all cell lines with suppressed mNT and/or NAF-1 (Fig 4), provide a strong link between the function of mNT and NAF-1 and the maintenance of labile iron levels in the mitochondria. Thus, both mNT and NAF-1 are required to maintain the levels of labile iron in the mitochondria under control and they do so by participating in the same pathway. It is of course possible that mNT and NAF-1 function in different pathways that affect each other indirectly and result in alterations in mitochondrial membrane potential, ROS and iron, and further studies are needed to dissect these interactions.

MitoNEET and NAF-1 were shown to donate their clusters to Anamorsin [19] and mNT is thought to repair Aconitase or possibly donate its clusters in a complex mechanism to Aconitase [20, 36], it is therefore possible that mNT and NAF-1 form a cluster transfer relay that mediates the transfer of clusters from the mitochondria to the cytosol (Fig 6). Any disruption in this relay by, for example, suppressing the levels of mNT or NAF-1 [4] or introducing a NAF-1 mutant that is unable to accept or donate its cluster to cancer cells [14], will therefore result in the over accumulation of labile iron in mitochondria and the subsequent over-accumulation of ROS (Fig 4; [4, 14]). The flow of Fe-S clusters from the mitochondria to the cytosol could therefore be used by cells to monitor mitochondrial function and regulate cellular proliferation, autophagy or apoptosis (Fig 6). As previously proposed, the regulation of autophagy and/or apoptosis could occur through interactions of NAF-1 with BCL-2 [3–5], and our RNA-Seq analysis of mNT(-) cells (S2 Table) and NAF-1(-) cells (4, 5), as well as work by others [47–49], suggest that the regulation of cellular proliferation by NAF-1 could occur through the MAPK and PI3K-Akt pathways (Fig 6).

Taken together, our results support a model in which mNT and NAF-1 cooperate to maintain the levels of labile Fe under control in the mitochondria. In addition to performing this function they may also serve as a link between the levels of Fe and ROS in the mitochondria and the regulation of cellular proliferation and/or apoptosis/autophagy activation (Fig 6). Based on the large difference between the number of transcripts altered in expression in NAF-1(-) and mNT(-) cells (Fig 5), it is possible that NAF-1 is also involved in mediating many other signals in cells and could even integrate mitochondrial function with these signals (Fig 6), whereas mNT may have a more limited function in cancer cells that is directly linked to Fe and Fe-S biogenesis/transport. Further studies are needed to dissect the different cellular pathways mediated by mNT and NAF-1 in cancer cells (Fig 5). Our study provides however evidence for a direct link between mNT and NAF-1 function in cancer cells and proposes that they regulate Fe/ROS/Fe-S levels in cells and tie them to the regulation of cellular proliferation and apoptosis/autophagy activation via protein-protein and cluster transfer reactions.

## Materials and methods

### Yeast two-hybrid

Yeast two-hybrid screening was performed by Hybrigenics Services, S.A.S., Paris, France (http://www.hybrigenics-services.com). The coding sequence for *Homo sapiens*-mitoNEET (aa 31–108) (GenBank accession number gi:374671792) was PCR-amplified and cloned into pB29 as an N-terminal fusion to LexA (N-mitoNEET-LexA-C). The construct was checked by sequencing the entire insert and used as a bait to screen a random-primed Human Normal Breast and a Human Breast Tumor Epithelial Cells cDNA library constructed into pP6. pB29 and pP6 are derived from the original pBTM116 [25, 26] and pGADGH plasmids, respectively. For the Human Normal Breast library, 59.4 million clones (6 fold the complexity of the library) were screened using a mating approach with YHGX13 (Y187 ade2-101::loxP-kanMX-loxP, matα) and L40ΔGal4 (mata) yeast strains as previously described [27]. 106 His+ colonies were selected on a medium lacking tryptophan, leucine and histidine. For the Human Breast Tumor Epithelial Cells library, 100 million clones (10 fold the complexity of the library) were screened using the same mating approach. 176 His+ colonies were selected on a medium lacking tryptophan, leucine and histidine. The prey fragments of the positive clones were amplified by PCR and sequenced at their 5’ and 3’ junctions. The resulting sequences were used to identify the corresponding interacting proteins in the GenBank database (NCBI) using a fully automated procedure. A confidence score (PBS, for Predicted Biological Score) was attributed to each interaction as previously described [28]. The provided Predicted Biological Score (PBS) represents the probability of an interaction being nonspecific. For practical use, the scores were divided into four categories, from A (highest confidence) to D (lowest confidence). PBS scores have been shown to positively correlate with the biological significance of interactions [29, 30].

### BiFC

The c-terminal (aa 175–239) and n-terminal (aa 1–174) fragments of YFP were PCR amplified from pEYFP-Pds1Δdestruction box (383) which was a gift from Jonathon Pines (Addgene plasmid # 39848), sequenced, and cloned as an in-frame fusion to pEGFP-N1 containing NAF-1 or mNT [19]. The linker sequence DPRSIAT was used for connecting the mNT/NAF-1 proteins to the YFPc/YFPn fragments. HEK-293 cells plated in EMEM (ATCC 30–2003) with 10% FBS on coverslips coated with collagen (A1048301, gibco) were co-transfected with the different fusion protein constructs (S1A Fig), using the GeneJuice Transfection Reagent (70967–3, Novagen; [4–6]). Following staining with the ER-Tracker Red Dye (E34250, Invitrogen) or MitoTracker Deep Red FM (M22426, Invitrogen), complete FluoroBrite DMEM (A18967-01, Gibco) medium was used to replace the RPMI medium 1640, and confocal images were obtained using a Zeiss LSM 710 confocal microscope. YFP, Mito-Tracker, ER-Tracker and DAPI were excited by a 488nm Argon, 561nm diode, 633nm diode and 405nm Diode laser respectively, and their fluorescence was detected at 508-604nm, 635-722nm, 585-700nm and 400-585nm, respectively.

### Iron-sulfur cluster transfer

NAF-1 and mNT proteins were purified as previously described [7–9, 31, 32]. Holo mNT and apo-NAF-1 were incubated in the presence or absence of 2% β-mercaptoethanol for 20 min. Transfer of the [2Fe–2S] cluster from mNT to NAF-1 was then visualized by native PAGE [6–9, 31] and confirmed by staining with Coomassie Blue (to verify the constant presence of both proteins during the cluster-transfer reaction). The red band visualized on the native gels indicates the presence of the 2Fe–2S cluster in the different (mNT/NAF-1) proteins. The native gel used to study the cluster transfer from mNT to NAF-1 was adapted from the previous studies of NAF-1 [31] with the following modifications; an upper stacking gel of 5% Polyacrylamide in 121.5 mM Tris-HCl pH 6.8 buffer was added, and the separating (lower) gel was changed to contain 15% Polyacrylamide in 375 mM Tris-HCl pH 8.8 buffer.

### Direct coupling analysis (DCA)

We used co-evolutionary constraints as well as the iron-sulfur proximity constraint to run a molecular dynamics simulation using structure based models [54] in gromacs [55]. mNT and NAF-1 were initially placed 75 Å apart, and the simulation was run using a C-alpha backbone representation of these proteins, with the constraints in place. The simulation was repeated 10 times, and all results converged to similar orientations, with slight differences in residue-residue distances. Of these 10 simulations, we selected the configuration that contained the fewest steric clashes. The resulting model served as an input for Fragment-Guided Molecular Dynamics Simulation (FG-MD), a molecular dynamics simulation algorithm that builds an optimized all-atom model from the C-alpha traces [56]. This tool eliminated steric clashes, and built a refined atomic-level model of the complex. The surface representation of this model is shown in Fig 3. To produce the C-alpha backbone structure in Fig 3, the PDB crystal structure of mNT (PDB 2QH7) and NAF-1 (PDB 3FNV) were aligned to the output of the FG-MD simulation (the final all-atom model of the complex), reinserting the iron-sulfur clusters in their respective locations within each protein. Since the crystal structures do not have exactly the same tertiary structure as the proteins in the final complex, this representation can vary from the actual model in some areas by around 1 angstrom. We aligned the PDB crystal structures to the final complex in order to show the distance between the iron-sulfur clusters. S4 Fig shows the model without the iron-sulfur clusters that is a direct output of the all-atomic reconstruction using FG-MD.

### shRNA suppression

MDA-MB-231 human epithelial breast cancer cells were grown in 37°C and 5% CO2 RPMI medium 1640 supplemented with 10% FCS and antibiotics (Biological industries). Plasmids containing shRNA for NAF-1 or mNT were in pGFP-RS vectors as previously described [4, 5, 14]. All plasmids were transfected using Lipofectamine 2000 (Invitrogen) or GeneJuice (EMD Millipore) as a transfection reagent according to manufacturer’s instructions. Double transfected cancer cells were treated with puromycin antibiotic 2ug/ml and blasticidin antibiotic with a concentration of 40ug/ml. Stable cell lines were obtained by FACS sorting based on GFP fluorescence and characterized for their mNT and/or NAF-1 proteins expression using protein blots as described previously [4, 5, 14].

### Mitochondrial labile iron (mLI), ROS and membrane potential (MMP)

Cells were cultured in glass-bottomed microscope dishes and analyzed using an epi-fluorescent microscope with a confocal (quality equivalent) opti-grid device (Nikon TE 2000 microscope equipped with a thermostatted stage and a Hamamatsu Orca-Era CCd camera) and driven by the Volocity 4 operating system (Improvision), which was used for both image data acquisition and analysis [4]. MMP was measured microscopically with TMRE (tetramethylrhodamine ethyl ester) using Texas Red (excitation 543nm, emission 633nm) filter sets, and the concentration was optimized depending on the cell lines used (0.05–0.5 μM) [4]. Mitochondrial iron accumulation was measured using RPA (rhodamine B-[(1,10-phenanthrolin-5-yl) aminocarbonyl] benzyl ester) using excitation 564nm, emission 603nm as described in [4]. Mitochondrial ROS production was determined using mitoSOX Red (Invitrogen, M36008), with excitation 510nm, and emission 580nm as described in [5]. DFP (ferriprox; 1,2-dimethyl-3-hydroxypyridin-4-one; Apo Pharma) was used as described in [4–6].

### RNA-Seq

Control (scrambled vector) and MCF-7 cells with suppressed mNT expression were grown and sampled side-by-side with MCF-7 cells with suppressed NAF-1 expression as described in [5]. RNA was extracted from cells using the Qiagen RNeasy Mini Kit (catalog number 74104). Three biological replicates were obtained each for control and mNT-suppressed cells (each biological replicate was obtained from a pool of three different plates and contained over five million cells each). Paired-end Illumina sequencing generated on average ∼20.9 M read pairs per sample, with each sequence read of a length of 101 nucleotides (http://www.biotech.wisc.edu/gcow). Bowtie was used for alignment of paired-end reads onto the human build-37.2 reference genome; Tophat was used for parsing the alignment to infer the exon—exon splice junctions, and Cufflinks was used to perform the differential expression analysis of annotated genes as described in [5]. The abundance of a transcript was measured in terms of ‘fragments per kb of transcript per million fragments mapped’ (FPKM), normalized for the transcript length and total number of cDNA fragments for a sample replicate. The raw sequence read datasets and expression results have been deposited to the NCBI GEO database repository (https://www.ncbi.nlm.nih.gov/geo/) and can be accessed with the accession number GSE87626.

### Statistical analysis

The statistical significance of the fold-change in transcript steady-state levels between two different conditions was assessed for RNA-Seq analysis based on a negative binomial model that was estimated from the data [5]. The fold-change in the transcription of genes with multiple isoforms was assessed by summing up the FPKMs for all isoforms of a gene and then measuring the difference in this under the two conditions [5]. The statistical significance test for protein expression, and analysis of epi-fluorescent microscope and confocal images was performed by using a one-tailed Student's t-test, as previously described [4–6]. Results are presented as mean±s.d. (*P<0.05; **P<0.01; ***P<0.001).

## Supporting information

S1 Fig Bar graphs showing the cell counts for co-localization of the BiFC signal with Mito or ER tracker in Fig 1. (PDF)

S2 Fig The top 20 DCA pairs (green) after filtering out pairs based on solvent accessibility, along with the known monomeric contacts (blue) and the experimentally determined dimeric contacts (red). The remaining top DCA pairs were predicted to be involved with the formation of a complex between mNT and NAF-1.
(PDF)

S3 Fig Images highlighting the region of the interface where a portion of mNT protrudes outward and fits into a similarly sized hole on the surface of NAF-1, following a lock-and-key configuration. The image on the left shows the portion of mNT that sticks out, while the image on the right is rotated, and shows the hole on the surface of NAF-1 where the piece from mNT is inserted. This part of the interface provides additional evidence to support the validity of our model.
(PDF)

S4 Fig The actual c-alpha backbone of the model, without aligning the PDB structures to it. Although the iron-sulfur clusters are not present, the distance between where they should be located is shown in red. The DCA couplings are depicted as green pseudobonds.
(PDF)

S1 Table A table showing the closest distance between each DCA pair. The first column lists the residue number for chain A of NAF-1 (PDB ID: 3FNV), and the second column lists the corresponding coupled residue for chain B of mNT (PDB ID: 2QH7). The last column indicates the minimum distance between these two residues in angstroms. The couplings are listed in order based on their DI values.
(PDF)

S2 Table Transcripts that overlap between NAF-1 and mNT. (PDF)

S3 Table Transcripts unique to mNT. (PDF)
