# Supplementary material for: Interactions between mitoNEET and NAF-1 in cells
Source: PLoS One. 2017 Apr 20;12(4):e0175796. doi: 10.1371/journal.pone.0175796 (PMC5398536; doi:10.1371/journal.pone.0175796)
Supplement: S1 Fig — (PDF) [file pone.0175796.s001.pdf]

Supplementary material for:

Interactions between mitoNEET and NAF-1 in cells

Ola Karmi[1,a], Sarah H. Holt[1,b], Luhua Song[1,b], Sagi Tamir[a], Yuting Luo[b], Ammar Adenwalla[c], Merav Darash-Yahana[a], Patricia A. Jennings[d], Rajeev K. Azad[b,e], Jose' N. Onuchic[f], Faruck Morcos[c], Rachel Nechushtai[2,a] and Ron Mittler[2,b]

[a]The Alexander Silberman Institute of Life Science and The Wolfson Institute for Applied Structural Biology, Hebrew University of Jerusalem, Edmond J. Safra Campus at Givat Ram, Jerusalem 91904, Israel. [b]Department of Biological Sciences and BioDiscovery Institute, University of North Texas, Denton TX 76203, USA. [c]Departments of Biological Sciences and Bioengineering, University of Texas at Dallas, 800 West Campbell Road, Richardson, TX 75080, USA. [d]Department of Chemistry & Biochemistry, University of California at San Diego, La Jolla, CA 92093, USA. [e]Department of Mathematics, University of North Texas, Denton, TX 76203, USA. [f]Center for Theoretical Biological Physics and Departments of Physics and Astronomy, Chemistry and Biosciences, 239 Brockman Hall, 6100 Main Street- MS-61, Rice University, Houston, TX 77005, USA.

Supplementary Figures:

Figure S1. Bar graphs showing the cell counts for co-localization of the BiFC signal with Mito or ER tracker in Figure 1.

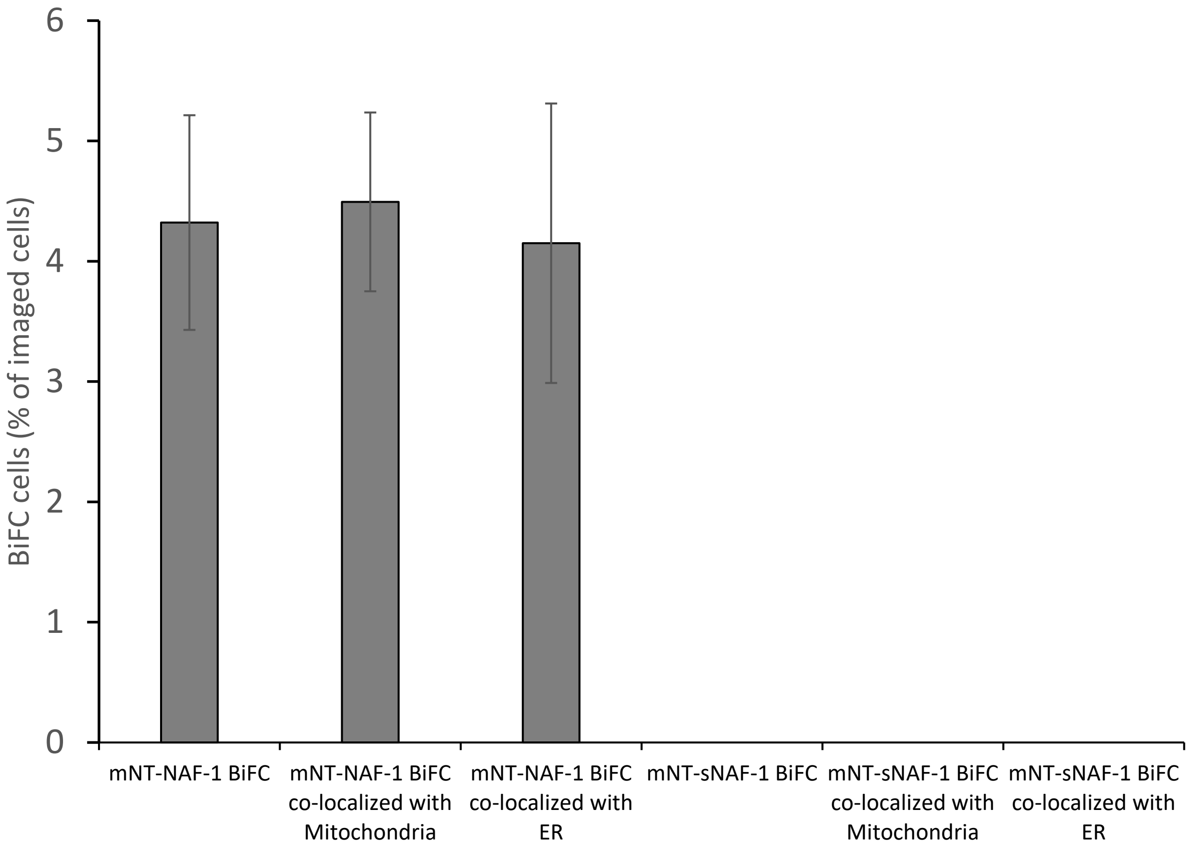

Figure S1. Bar graphs showing the cell counts for co-localization of the BiFC signal with Mito or ER tracker in Figure 1.
