# Supplementary material for: Interactions between mitoNEET and NAF-1 in cells
Source: PLoS One. 2017 Apr 20;12(4):e0175796. doi: 10.1371/journal.pone.0175796 (PMC5398536; doi:10.1371/journal.pone.0175796)
Supplement: S3 Fig — The image on the left shows the portion of mNT that sticks out, while the image on the right is rotated, and shows the hole on the surface of NAF-1 where the piece from mNT is inserted. This part of the interface provides additional evidence to support the validity of our model. (PDF) [file pone.0175796.s003.pdf]

Supplementary material for:

Interactions between mitoNEET and NAF-1 in cells

Ola Karmi[1,a], Sarah H. Holt[1,b], Luhua Song[1,b], Sagi Tamir[a], Yuting Luo[b], Ammar Adenwalla[c], Merav Darash-Yahana[a], Patricia A. Jennings[d], Rajeev K. Azad[b,e], Jose' N. Onuchic[f], Faruck Morcos[c], Rachel Nechushtai[2,a] and Ron Mittler[2,b]

[a]The Alexander Silberman Institute of Life Science and The Wolfson Institute for Applied Structural Biology, Hebrew University of Jerusalem, Edmond J. Safra Campus at Givat Ram, Jerusalem 91904, Israel. [b]Department of Biological Sciences and BioDiscovery Institute, University of North Texas, Denton TX 76203, USA. [c]Departments of Biological Sciences and Bioengineering, University of Texas at Dallas, 800 West Campbell Road, Richardson, TX 75080, USA. [d]Department of Chemistry & Biochemistry, University of California at San Diego, La Jolla, CA 92093, USA. [e]Department of Mathematics, University of North Texas, Denton, TX 76203, USA. [f]Center for Theoretical Biological Physics and Departments of Physics and Astronomy, Chemistry and Biosciences, 239 Brockman Hall, 6100 Main Street- MS-61, Rice University, Houston, TX 77005, USA.

Supplementary Figures:

Figure S3. Images highlighting the region of the interface where a portion of mNT protrudes outward and fits into a similarly sized hole on the surface of NAF-1, following a lock-and-key configuration. The image on the left shows the portion of mNT that sticks out, while the image on the right is rotated, and shows the hole on the surface of NAF-1 where the piece from mNT is inserted. This part of the interface provides additional evidence to support the validity of our model.

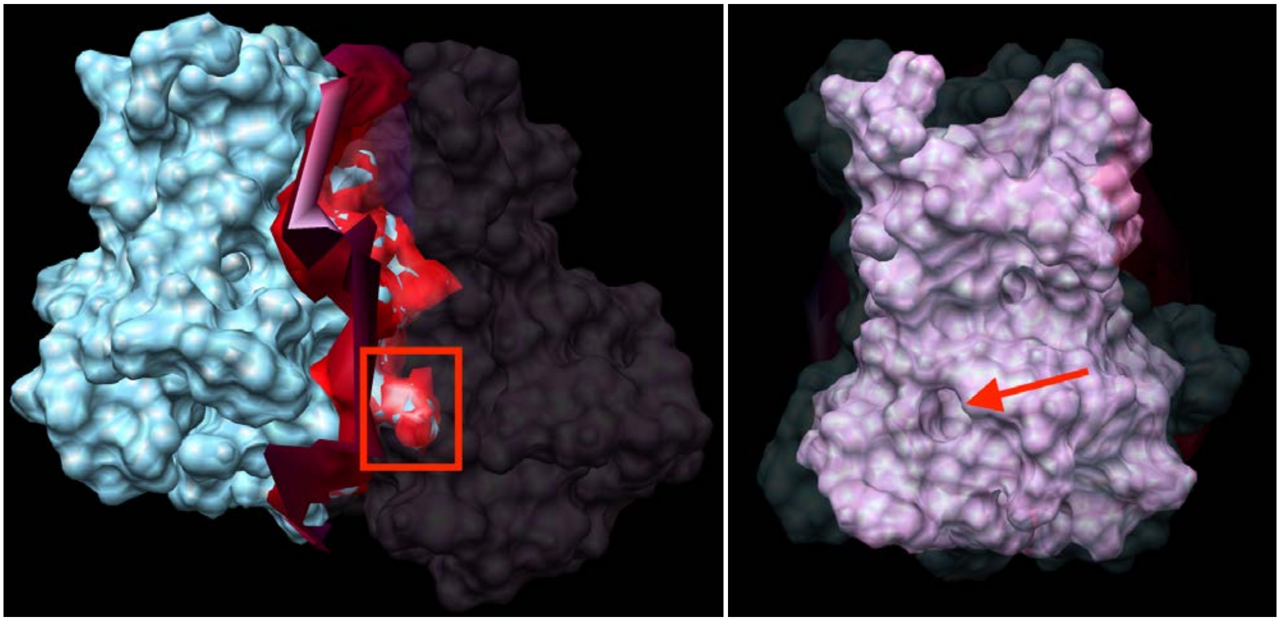

Figure S3. Images highlighting the region of the interface where a portion of mNT protrudes outward and fits into a similarly sized hole on the surface of NAF-1, following a lock-and-key configuration. The image on the left shows the portion of mNT that sticks out, while the image on the right is rotated, and shows the hole on the surface of NAF-1 where the piece from mNT is inserted. This part of the interface provides additional evidence to support the validity of our model.
