# Supplementary material for: Interactions between mitoNEET and NAF-1 in cells
Source: PLoS One. 2017 Apr 20;12(4):e0175796. doi: 10.1371/journal.pone.0175796 (PMC5398536; doi:10.1371/journal.pone.0175796)
Supplement: S1 Table — The first column lists the residue number for chain A of NAF-1 (PDB ID: 3FNV), and the second column lists the corresponding coupled residue for chain B of mNT (PDB ID: 2QH7). The last column indicates the minimum distance between these two residues in angstroms. The couplings are listed in order based on their DI values. (PDF) [file pone.0175796.s005.pdf]

Supplementary material for:

Interactions between mitoNEET and NAF-1 in cells

Ola Karmi[1,a], Sarah H. Holt[1,b], Luhua Song[1,b], Sagi Tamir[a], Yuting Luo[b], Ammar Adenwalla[c], Merav Darash-Yahana[a], Patricia A. Jennings[d], Rajeev K. Azad[b,e], Jose' N. Onuchic[f], Faruck Morcos[c], Rachel Nechushtai[2,a] and Ron Mittler[2,b]

[a]The Alexander Silberman Institute of Life Science and The Wolfson Institute for Applied Structural Biology, Hebrew University of Jerusalem, Edmond J. Safra Campus at Givat Ram, Jerusalem 91904, Israel. [b]Department of Biological Sciences and BioDiscovery Institute, University of North Texas, Denton TX 76203, USA. [c]Departments of Biological Sciences and Bioengineering, University of Texas at Dallas, 800 West Campbell Road, Richardson, TX 75080, USA. [d]Department of Chemistry & Biochemistry, University of California at San Diego, La Jolla, CA 92093, USA. [e]Department of Mathematics, University of North Texas, Denton, TX 76203, USA. [f]Center for Theoretical Biological Physics and Departments of Physics and Astronomy, Chemistry and Biosciences, 239 Brockman Hall, 6100 Main Street- MS-61, Rice University, Houston, TX 77005, USA.

Supplementary Tables:

Table S1. A table showing the closest distance between each DCA pair. The first column lists the residue number for chain A of NAF-1 (PDB ID: 3FNV), and the second column lists the corresponding coupled residue for chain B of mNT (PDB ID: 2QH7). The last column indicates the minimum distance between these two residues in angstroms. The couplings are listed in order based on their DI values.

NAF-1 3FNV Chain A	mNT 2QH7 Chain B	Distance (Å)
87	69	7.36
80	87	13.00
87	70	4.35
90	78	10.14
80	70	22.00
90	79	6.40
89	78	13.61
81	69	22.88
80	74	18.52
89	79	8.28
87	76	13.41
87	78	11.46
81	76	16.41
85	88	5.77
90	88	14.57
88	70	7.58
90	73	11.13
87	86	9.22
87	85	6.57
86	88	7.92
**Average**		11.53

Table S1. A table showing the closest distance between each DCA pair. The first column lists the residue number for chain A of NAF-1 (PDB ID: 3FNV), and the second column lists the corresponding coupled residue for chain B of mNT (PDB ID: 2QH7). The last column indicates the minimum distance between these two residues in angstroms. The couplings are listed in order based on their DI values.
