# Supplementary material for: Interactions between mitoNEET and NAF-1 in cells
Source: PLoS One. 2017 Apr 20;12(4):e0175796. doi: 10.1371/journal.pone.0175796 (PMC5398536; doi:10.1371/journal.pone.0175796)
Supplement: S2 Table — (PDF) [file pone.0175796.s006.pdf]

Supplementary material for:

Interactions between mitoNEET and NAF-1 in cells

Ola Karmi[1,a], Sarah H. Holt[1,b], Luhua Song[1,b], Sagi Tamir[a], Yuting Luo[b], Ammar Adenwalla[c], Merav Darash-Yahana[a], Patricia A. Jennings[d], Rajeev K. Azad[b,e], Jose' N. Onuchic[f], Faruck Morcos[c], Rachel Nechushtai[2,a] and Ron Mittler[2,b]

[a]The Alexander Silberman Institute of Life Science and The Wolfson Institute for Applied Structural Biology, Hebrew University of Jerusalem, Edmond J. Safra Campus at Givat Ram, Jerusalem 91904, Israel. [b]Department of Biological Sciences and BioDiscovery Institute, University of North Texas, Denton TX 76203, USA. [c]Departments of Biological Sciences and Bioengineering, University of Texas at Dallas, 800 West Campbell Road, Richardson, TX 75080, USA. [d]Department of Chemistry & Biochemistry, University of California at San Diego, La Jolla, CA 92093, USA. [e]Department of Mathematics, University of North Texas, Denton, TX 76203, USA. [f]Center for Theoretical Biological Physics and Departments of Physics and Astronomy, Chemistry and Biosciences, 239 Brockman Hall, 6100 Main Street- MS-61, Rice University, Houston, TX 77005, USA.

Supplementary Tables:

Table S2. Transcripts that overlap between NAF-1 and mNT

Table S2. Transcripts that overlap between NAF-1 and mNT

Gene_id	Annotation	Locus	value_1	value_2	Log2(fold_change)	test_stat	p_value	q_value
COL21A1	collagen alpha-1(XXI) chain	6:55921387-56112378	0.665505	0.038562	-4.1092	4.26865	1.97E-05	0.00296344
A2M	alpha-2-macroglobulin	12:9217772-9268558	1.53042	0.0918392	-4.05867	6.12191	9.25E-10	7.23E-07
KCNH7	potassium voltage-gated channel, subfamily H (eag-related), member 7	2:163227916-163695257	0.98606	0.0807677	-3.60983	4.75553	1.98E-06	0.000412584
CALCR	calcitonin receptor	7:93053798-93204042	6.04123	0.788253	-2.93811	9.96363	0	0
KLK11	kallikrein-related peptidase 11	19:51525486-51531290	15.5583	2.15511	-2.85185	8.15627	4.44E-16	7.93E-13
TRIP6	thyroid hormone receptor interactor	7:100464949-100471076	1.58967	0.249258	-2.67302	4.24883	2.15E-05	0.00316198
NECAB1	N-terminal EF-hand calcium binding protein 1	8:91803920-91997485	3.83994	0.655031	-2.55145	8.80958	0	0
DPYD	dihydropyrimidine dehydrogenase	1:97543298-98386615	2.67358	0.462752	-2.53046	4.64759	3.36E-06	0.000636405
ARHGAP36	Rho GTPase activating protein 36	X:130192215-130223857	1.49902	0.265334	-2.49814	5.55295	2.81E-08	1.21E-05
SLFN5	schlafen family member 5	17:33570085-33594768	0.900658	0.166309	-2.43711	5.31594	1.06E-07	3.40E-05
AREG	amphiregulin	4:75310852-75320726	29.1358	5.9155	-2.30022	8.36863	0	0
PRSS23	seerine protease 23	11:86511490-86522275	164.676	34.4925	-2.25527	10.7934	0	0
ITGB8	integrin beta-8	7:20370724-20455382	1.18098	0.250419	-2.23757	7.17351	7.31E-13	1.02E-09
NPY5R	neuropeptide Y receptor Y5	4:164265090-164273086	3.0232	0.648336	-2.22126	5.73779	9.59E-09	4.44E-06
ANKRD35	ankyrin repeat domain-containing protein	1:145549208-145568526	0.83891	0.189215	-2.14849	4.15234	3.29E-05	0.0045733
CFB	complement factor B	6:31913720-31919861	3.90114	0.948321	-2.04045	6.63155	3.32E-11	3.78E-08
AREGB	amphiregulin B	4:75480838-75490481	34.8491	8.58735	-2.02084	6.21326	5.19E-10	4.33E-07
LOC100507584		6:33857287-33864684	3.35472	0.848163	-1.98378	6.57956	4.72E-11	4.92E-08
CAPN8	calpain-8	1:223714971-223853436	4.14538	1.07415	-1.94831	5.89855	3.67E-09	2.18E-06
ABCC2	ATP-binding cassette, sub-family C (CFTR/MRP), member 2	10:101542462-101611662	1.29275	0.357023	-1.85636	5.49072	4.00E-08	1.62E-05
S100A9	S100 calcium binding protein A9 (calgranulin B	1:153330329-153333503	23.0307	6.66354	-1.7892	5.85892	4.66E-09	2.51E-06
NPY1R	neuropeptide Y receptor type 1	4:164245116-164253947	135.032	39.4299	-1.77595	8.28729	2.22E-16	4.63E-13
HSD17B11	17-beta-hydroxysteroid dehydrogenase 11	4:88257690-88312455	2.95682	0.867262	-1.7695	4.93314	8.09E-07	0.000194626
SLITRK6	SLIT and NTRK-like protein 6	13:86366921-86373483	53.2272	15.7042	-1.76102	8.07382	6.66E-16	1.04E-12
NCF2	neutrophil cytosolic factor 2	1:183524696-183560056	2.05444	0.621187	-1.72565	4.4182	9.95E-06	0.00161662
APCDD1	adenomatosis polyposis coli down-regulated 1 protein	18:10454624-10488698	1.2891	0.397516	-1.69728	3.98934	6.63E-05	0.00843768
MACC1	metastasis-associated in colon cancer protein 1	7:20174277-20257013	1.10165	0.363343	-1.60026	5.42869	5.68E-08	2.03E-05
SOX2	SRY (sex determining region Y)-box 2	3:181328150-181459009	6.07716	2.00584	-1.59919	5.85345	4.81E-09	2.51E-06
SIGLEC15	sialic acid binding Ig-like lectin 15	18:43405544-43422521	6.13647	2.0293	-1.59643	5.10518	3.30E-07	8.61E-05
SYT10	synaptotagmin-10	12:33528347-33592754	0.840082	0.281489	-1.57745	3.542	0.000397099	0.0385001
C3	complement component C3	19:6677845-6720662	1.21008	0.405479	-1.5774	4.78314	1.73E-06	0.000365842
HMCN1	hemicentin-1	1:185703682-186160085	0.58194	0.195794	-1.57154	5.52845	3.23E-08	1.35E-05
LOC100506351		8:91658668-91663985	0.874354	0.299553	-1.54541	3.63054	0.000282833	0.0291487
IFITM1	interferon-induced transmembrane protein 1	11:313990-315272	6.48628	2.26649	-1.51693	3.86164	0.000112627	0.0131647
IGSF1	immunoglobulin superfamily member 1	X:130407479-130423403	38.9458	13.6528	-1.51227	5.07152	3.95E-07	0.000100731
FIGN	fidgetin	2:164464117-164592513	2.41038	0.8464	-1.50985	5.36923	7.91E-08	2.75E-05
NAV2	neuron navigator 2	11:19372270-20143147	2.81207	0.988318	-1.50858	5.01918	5.19E-07	0.000129806
PKIA	protein kinase (cAMP-dependent, catalytic) inhibitor alpha	8:79428335-79517502	2.04679	0.728786	-1.4898	4.73495	2.19E-06	0.000449255
ARSJ	arylsulfatase J	4:114821439-114900878	5.78402	2.08462	-1.47229	5.93645	2.91E-09	1.82E-06
PAPSS2	3'-phosphoadenosine 5'-phosphosulfate synthase 2	10:89419475-89507462	8.88142	3.22139	-1.46311	6.04628	1.48E-09	1.09E-06
PMP22	peripheral myelin protein 22	17:15133095-15168644	8.84072	3.25008	-1.44369	3.97837	6.94E-05	0.00859249
CDH18	cadherin-18	5:19473139-19988353	8.2274	3.0377	-1.43745	5.77615	7.64E-09	3.68E-06
ADCY1	adenylate cyclase type I	7:45614124-45762715	13.5108	5.18359	-1.38209	6.36365	1.97E-10	1.76E-07
CAV1	caveolin-1	7:116164838-116201239	9.87606	3.79893	-1.37834	4.25183	2.12E-05	0.00315693
SYTL5	synaptotagmin-like 5	X:37865834-37988073	30.6603	11.9393	-1.36065	6.01123	1.84E-09	1.21E-06
FHL1	four and a half LIM domains protein 1	X:135228860-135293518	29.7615	11.692	-1.34793	5.36049	8.30E-08	2.81E-05
FSTL4	Follistatin-like protein 4	5:132532151-132948223	0.820848	0.324687	-1.33806	3.71031	0.000207009	0.022912
SERPINA3	serpin peptidase inhibitor, clade A (alpha-1 antiproteinase, antitrypsin), member 3	14:95078713-95090390	233.222	92.5004	-1.33417	6.38561	1.71E-10	1.64E-07
SGK1	serum/glucocorticoid regulated kinase 1	6:134490383-134639196	4.29007	1.71328	-1.32424	4.57745	4.71E-06	0.000853153
APOL6	apolipoprotein L6	22:36044423-36064456	0.709893	0.286433	-1.3094	4.34493	1.39E-05	0.00223391
OLFML3	olfactomedin-like protein 3	1:114522029-114524875	24.8045	10.0132	-1.3087	5.47806	4.30E-08	1.68E-05
TMEM64	transmembrane protein 64	8:91634222-91658133	92.5456	37.3853	-1.30769	6.03048	1.63E-09	1.14E-06
KCNJ8	Potassium channel, inwardly rectifying subfamily J member 8	12:21917888-21927755	10.7166	4.35019	-1.30069	5.21227	1.87E-07	5.23E-05
DKK1	dickkopf-1 like	10:54074040-54077417	66.0224	26.9134	-1.29463	5.84111	5.19E-09	2.59E-06
UGT2B15	UDP glucuronosyltransferase 2 family, polypeptide B15	4:69512314-69536494	20.8714	8.53592	-1.28991	5.44142	5.29E-08	1.94E-05
TFPI2	tissue factor pathway inhibitor 2	7:93514708-93520303	15.8859	6.57832	-1.27196	5.29101	1.22E-07	3.66E-05
MUCL1	mucin-like protein 1	12:55248298-55252177	49.2851	20.4797	-1.26696	4.78321	1.73E-06	0.000365842
ATRNL1	attractin-like protein 1	10:116853123-117708496	1.40096	0.585653	-1.2583	4.63618	3.55E-06	0.000662511
C5orf4	fatty acid hydroxylase domain containing 2	5:154198051-154230213	16.5454	6.98906	-1.24326	5.29093	1.22E-07	3.66E-05
GRB14	growth factor receptor-bound protein 14	2:165349322-165478360	5.37517	2.27482	-1.24056	4.55764	5.17E-06	0.000911259
FHL2	four and a half LIM domains protein 2	2:105977282-106055230	8.0556	3.41302	-1.23895	3.58027	0.000343239	0.0340966
RUNX1	SL3-3 enhancer factor 1 alpha B subunit	21:36160097-36421595	12.6272	5.38847	-1.22858	4.55786	5.17E-06	0.000911259
GPRC5A	G protein-coupled receptor, family C, group 5, member A	12:13043955-13066600	32.6877	13.9498	-1.2285	5.44348	5.22E-08	1.94E-05
CAPN13	Calcium-activated neutral proteinase 13	2:30945636-31030311	3.67926	1.59366	-1.20707	4.29472	1.75E-05	0.00270078
LRP1B	low density lipoprotein receptor-related protein 1B	2:140988995-142889270	0.758863	0.32883	-1.2065	4.54406	5.52E-06	0.00095856
SPTSSB	serine palmitoyltransferase, small subunit B	3:161062579-161089871	540.08	237.117	-1.18757	5.86284	4.55E-09	2.51E-06
CLDN9	claudin 9	16:3062456-3064506	4.06264	1.78708	-1.18482	3.98342	6.79E-05	0.00849599
PGM2L1	Phosphoglucomutase-2-like 1	11:74041360-74109502	2.31438	1.02398	-1.17644	4.50208	6.73E-06	0.00113731
LGALS3BP	lectin, galactoside-binding, soluble, 3 binding protein	17:76967334-76976061	20.4934	9.35337	-1.1316	4.73156	2.23E-06	0.000449452
CADM1	Synaptic cell adhesion molecule	11:115044344-115375241	2.39182	1.09363	-1.12899	3.46367	0.000532868	0.0482941
SLC6A14	solute carrier family 6 (neurotransmitter transporter), member 14	X:115567746-115592625	65.2853	30.121	-1.11599	5.28902	1.23E-07	3.66E-05
BMPER	Bone morphogenetic protein-binding endothelial cell precursor-derived regulator	7:33944522-34195484	4.85204	2.2866	-1.08539	4.43865	9.05E-06	0.0014897
UGDH	uridine diphospho-glucose dehydrogenase	4:39500374-39529218	145.812	69.5168	-1.06868	5.13815	2.77E-07	7.38E-05
NRSN2	neurensin-2	20:327369-335512	3.54672	1.69607	-1.06429	3.69166	0.000222792	0.0240212
CDK6	Cell division protein kinase 6	7:92234234-92465941	1.52927	0.736882	-1.05334	4.10941	3.97E-05	0.00539259
CDSN	corneodesmosin	6:31082607-31107869	6.09489	3.04434	-1.00147	3.81755	0.000134786	0.0151871
SCIN	scinderin	7:12610202-12693228	31.1239	15.7933	-0.978715	4.44663	8.72E-06	0.00145459
ELOVL2	Very-long-chain 3-oxoacyl-CoA synthase 2	6:10980991-11079377	192.78	98.2776	-0.972025	4.84602	1.26E-06	0.000286433
VAV3	vav 3 guanine nucleotide exchange factor	1:108113781-108537229	48.9065	25.3432	-0.948429	4.28879	1.80E-05	0.00274012
HEY2	hairy and enhancer of split-related protein 2	6:126070731-126082415	8.70823	4.52895	-0.943204	3.84282	0.000121627	0.0139559
ABHD2	alpha/beta hydrolase domain containing protein 2	15:89631380-89745591	29.0246	15.4709	-0.907719	4.3199	1.56E-05	0.00244041
SULF1	sulfatase 1	8:70378858-70573147	35.6523	19.0111	-0.907155	3.79001	0.000150642	0.0168222
WISP2	WNT1 inducible signaling pathway protein 2	20:43343884-43356452	14.5869	7.79034	-0.904914	3.47234	0.000515947	0.0471018
MAP3K1	MAP/ERK kinase kinase 1	5:56110899-56191979	15.5897	8.37749	-0.896006	4.1147	3.88E-05	0.00532821
PGR	progesterone receptor	11:100900354-101030001	14.6236	7.86875	-0.894092	3.90352	9.48E-05	0.0112923
NAALADL2	N-acetylated alpha-linked acidic dipeptidase-like 2	3:174577110-175523428	20.9458	11.3202	-0.887764	4.02309	5.74E-05	0.00756201
SLC16A1	solute carrier family 16 (monocarboxylic acid transporters), member 1	1:113454468-113498975	5.7755	3.12768	-0.884854	3.59219	0.000327909	0.0332414

SFXN2	sideroflexin-2	10:104474297-104498946	85.4667	46.2901	-0.88466	4.2144	2.50E-05	0.0036422
KLF5	Krueppel-like factor 5	13:73633141-73651676	9.09291	5.06073	-0.845397	3.48912	0.000484622	0.0455727
RPS6KA3	ribosomal protein S6 kinase alpha-3	X:20168028-20284750	5.54047	3.0978	-0.838764	3.57124	0.000355291	0.0347159
PDZK1	PDZ domain containing 1	1:145727665-145764207	90.8977	52.9224	-0.780365	3.57367	0.00035201	0.034666
CACNG4	Neuronal voltage-gated calcium channel gamma-4 subunit	17:64960979-65029518	60.6737	35.6137	-0.768637	3.62917	0.000284332	0.0291487
RAB31	ras-related protein Rab-31	18:9708227-9862553	84.8075	51.7897	-0.711527	3.49574	0.000472742	0.0447923
DHRS2	dehydrogenase/reductase (SDR family) member 2	14:24105572-24114848	416.274	675.629	0.698699	-3.66088	0.000251347	0.0266407
SLC7A5	solute carrier family 7 (cationic amino acid transporter, y+ system), member 5	16:87863628-87903100	137.968	225.577	0.709286	-3.6985	0.00021688	0.0237819
RAP1GAP	RAP1, GTPase activating protein 1	1:21922707-21995856	22.0111	39.1692	0.831487	-3.69641	0.000218671	0.0237819
CDKN1A	cyclin-dependent kinase inhibitor 1	6:36644236-36655116	79.066	142.529	0.850126	-4.15356	3.27E-05	0.0045733
ID3	inhibitor of DNA binding 3, dominant negative helix-loop-helix protein	1:23884420-23886285	16.093	29.1846	0.858772	-3.59087	0.00032957	0.0332414
CLGN	calmegin	4:141309606-141348815	7.66678	14.045	0.873361	-3.67847	0.000234641	0.0250825
CYP4F22	cytochrome P450, family 4, subfamily F, polypeptide 22	19:15619335-15663128	29.252	55.1254	0.914183	-4.33211	1.48E-05	0.00233814
RPS17L	40S ribosomal protein S17-like	15:83205503-83209208	485.357	957.262	0.979867	-4.87233	1.10E-06	0.000255446
IL20	interleukin-20	1:207039153-207042568	29.69	60.165	1.01895	-4.61601	3.91E-06	0.000719511
ACACB	acetyl-Coenzyme A carboxylase beta	12:109577201-109706031	0.375477	0.78312	1.06051	-3.5229	0.00042686	0.0410672
CYP1A1	cytochrome P450, family 1, subfamily A, polypeptide 1	15:75011882-75017877	5.25068	11.2477	1.09905	-4.52189	6.13E-06	0.00105005
GDF15	growth/differentiation factor 15	19:18496967-18499986	30.9424	69.0639	1.15835	-5.21048	1.88E-07	5.23E-05
DUSP2	dual specificity protein phosphatase 2	2:96808907-96811179	7.28059	16.3824	1.17002	-4.64944	3.33E-06	0.000636405
PLS3	plastin-3	X:114795176-114885181	1.49459	3.67884	1.2995	-3.90831	9.29E-05	0.0111773
SNCG	synuclein, gamma (breast cancer-specific protein 1)	10:88718287-88723017	9.39084	23.5198	1.32455	-4.90958	9.13E-07	0.000215379
ADRBK2	beta-adrenergic receptor kinase 2	22:25960860-26125259	0.66295	1.67096	1.33371	-4.97239	6.61E-07	0.000162176
FGFR4	fibroblast growth factor receptor 4	5:176513920-176525127	6.75855	17.1084	1.33992	-5.15661	2.51E-07	6.84E-05
GHR	growth hormone receptor	5:42423876-42721980	3.06653	7.91778	1.36849	-4.83347	1.34E-06	0.000299658
TM4SF1	transmembrane 4 L6 family member 1	3:149086804-149095568	1.10042	2.89087	1.39345	-3.82033	0.000133273	0.0151531
CHRM1	cholinergic receptor, muscarinic 1	11:62676150-62689012	0.551029	1.59049	1.52927	-4.01468	5.95E-05	0.00774744
ASTL	astacin-like metalloendopeptidase (M12 family)	2:96789588-96804175	1.26383	3.76562	1.57508	-4.10471	4.05E-05	0.00544423
PTGES	prostaglandin E synthase	9:132500614-132515344	2.25642	6.96821	1.62675	-5.56115	2.68E-08	1.20E-05
CRAT	Carnitine acetyltransferase	9:131857072-131873070	3.11253	10.6228	1.77101	-6.68933	2.24E-11	2.80E-08
