# Supplementary material for: Interactions between mitoNEET and NAF-1 in cells
Source: PLoS One. 2017 Apr 20;12(4):e0175796. doi: 10.1371/journal.pone.0175796 (PMC5398536; doi:10.1371/journal.pone.0175796)
Supplement: S3 Table — (PDF) [file pone.0175796.s007.pdf]

Supplementary material for:

Interactions between mitoNEET and NAF-1 in cells

Ola Karmi[1,a], Sarah H. Holt[1,b], Luhua Song[1,b], Sagi Tamir[a], Yuting Luo[b], Ammar Adenwalla[c], Merav Darash-Yahana[a], Patricia A. Jennings[d], Rajeev K. Azad[b,e], Jose' N. Onuchic[f], Faruck Morcos[c], Rachel Nechushtai[2,a] and Ron Mittler[2,b]

[a]The Alexander Silberman Institute of Life Science and The Wolfson Institute for Applied Structural Biology, Hebrew University of Jerusalem, Edmond J. Safra Campus at Givat Ram, Jerusalem 91904, Israel. [b]Department of Biological Sciences and BioDiscovery Institute, University of North Texas, Denton TX 76203, USA. [c]Departments of Biological Sciences and Bioengineering, University of Texas at Dallas, 800 West Campbell Road, Richardson, TX 75080, USA. [d]Department of Chemistry & Biochemistry, University of California at San Diego, La Jolla, CA 92093, USA. [e]Department of Mathematics, University of North Texas, Denton, TX 76203, USA. [f]Center for Theoretical Biological Physics and Departments of Physics and Astronomy, Chemistry and Biosciences, 239 Brockman Hall, 6100 Main Street- MS-61, Rice University, Houston, TX 77005, USA.

Supplementary Tables:

Table S3. Transcripts unique to mNT

Table S3. Transcripts unique to mNT

Gene_id	Annotation	Locus	value_1	value_2	Log2(fold_change)	test_stat	p_value	q_value
TCN1	transcobalamin I (vitamin B12 binding protein, R binder family	11:59620280-59634041	1.50342	0.232549	-2.69264	3.8785	0.000105102	0.012401
TLR2	toll-like receptor 2	4:154605440-154627243	0.901698	0.258577	-1.80205	4.01247	6.01E-05	0.00774744
EREG	epiregulin	4:75230859-75254477	0.609871	0.190532	-1.67847	3.65191	0.000260299	0.0273577
KRT23	keratin-23	17:39078951-39093836	1.84882	0.595015	-1.63561	4.20308	2.63E-05	0.00378531
ZC3H12C	Zinc finger CCCH domain-containing protein 12C	11:109964086-110042566	0.666939	0.25833	-1.36834	4.19792	2.69E-05	0.00382848
DOCK10	dedicator of cytokinesis 10	2:225629806-225907330	2.65852	1.15186	-1.20666	4.80563	1.54E-06	0.000338489
SERPINA5	acrosomal serine protease inhibitor	14:95047705-95059457	14.3004	6.53562	-1.12966	4.65913	3.18E-06	0.000620546
EPAS1	hypoxia-inducible factor 2 alpha	2:46524540-46613842	4.27982	2.13081	-1.00615	4.09236	4.27E-05	0.00568152
TUBB3	tubulin, beta 3	16:89988416-90002505	18.0217	9.21373	-0.967879	3.90863	9.28E-05	0.0111773
LCN2	neutrophil gelatinase-associated lipocalin	9:130911731-130915734	22.999	12.2719	-0.906209	3.48316	0.000495535	0.0462512
ZDHHC22	zinc finger, DHHC domain containing 22	14:77597612-77608134	10.445	5.88329	-0.828115	3.47732	0.000506457	0.0469205
CELSR2	Epidermal growth factor-like protein 2	1:109792640-109818378	57.7862	35.0755	-0.720262	3.58007	0.000343501	0.0340966
RPS29	40S ribosomal protein S29	14:50044038-50053094	2786.06	4683.53	0.74937	-3.64583	0.000266534	0.0277795
TGFB3	transforming growth factor beta-3	14:76424441-76448092	5.2145	9.24064	0.825464	-3.47455	0.000511706	0.0470581
C15orf59	chromosome 15 open reading frame 59	15:74032140-74043816	3.8681	7.78477	1.00903	-3.51915	0.000432928	0.041333
ARHGDIB	Rho GDP dissociation inhibitor (GDI) beta	12:15094949-15114562	4.82279	10.4317	1.11304	-3.98744	6.68E-05	0.00843768
FSIP1	fibrous sheath interacting protein 1	15:39892231-40075039	1.62949	3.59601	1.14198	-3.95377	7.69E-05	0.00943306
C10orf116	adipose-specific protein 2	10:88728187-88769960	26.3318	66.6485	1.33976	-5.3366	9.47E-08	3.12E-05
EAF2	ELL associated factor 2	3:121554033-121605373	1.77018	4.86015	1.45711	-3.84375	0.000121169	0.0139559
AKAP12	A kinase (PRKA) anchor protein 12	6:151561133-151679694	0.122096	4.22976	5.11448	-12.4586	0	0

CISD1	mitoNEET	10:60028861-60049019	7.95929	3.45614	-1.20348	4.6629	3.12E-06	0.000618973
